# Oral uracil treatment increases susceptibility to rectal SIV infection

**DOI:** 10.1371/journal.ppat.1014716

**Published:** 2026-10-22

**Authors:** Elizabeth G. Bodykevich, Fabiola Castello Casta, Jacob K. Flynn, Charlotte A. Nichols, Glenn A. Nardone, Christine M. Fennessey, Kelsie Brooks, Delmy Ruiz, Jennifer Simpson, Carol L. Vinton, Brandon F. Keele, Jason M. Brenchley, Alexandra M. Ortiz

**Affiliations:** 1 Barrier Immunity Section, Laboratory of Viral Diseases, Division of Intramural Research, National Institute of Allergy and Infectious Diseases (NIAID), National Institutes of Health (NIH), Bethesda, Maryland, United States of America; 2 Proteins and Chemistry Section, Research Technologies Branch, NIAID, NIH, Bethesda, Maryland, United States of America; 3 AIDS and Cancer Virus Program, Frederick National Laboratory for Cancer Research, Frederick, Maryland, United States of America; Duke University, UNITED STATES OF AMERICA

## Abstract

The intestinal microbiomes of men who have sex with men (MSM) are distinct as compared to non-MSM and may increase susceptibility to rectal HIV infection. We recently demonstrated that dysbiosis-associated disruptions to host antimicrobial immunity increase macaque susceptibility to rectal SIVmac239X acquisition. The described susceptibility-associated network identified Dual Oxidase 2 (DUOX2) – an epithelial sensor that catalyzes reactive oxygen species (ROS) production upon sensing pathobiont-derived uracil – as a likely contributor to increased SIV susceptibility. We hypothesized that intestinal, uracil bioavailability increases susceptibility to rectal HIV acquisition. Here, we treated rhesus macaques with oral uracil or uracil suppositories, prior to low-dose intra-rectal challenge with SIVmac239X. Oral treatment reduced pro-inflammatory cytokine production from peripheral T-cells but did not perturb rectal immunologic transcripts or epithelial ROS production and did not alter biomarkers of intestinal damage. Although treatment with uracil suppositories led to increased concentrations of fecal uracil and indices of intestinal damage, there were fewer immunological perturbations relative to oral treatment. Despite limited evidence of intestinal damage, oral uracil treatment increased susceptibility to intra-rectal SIV acquisition whereas uracil suppositories did not. These data provide mechanistic insights into intra-rectal SIV acquisition and may lead to novel therapeutic interventions that reduce incidence of HIV acquisition.

## Author summary

The intestinal microbiome is increasingly recognized as an important contributor to susceptibility to HIV infection, but the mechanisms underlying this relationship remain poorly understood. We previously identified changes in antimicrobial immune pathways associated with increased rectal SIV acquisition, including pathways responsive to the metabolite uracil. Here, we tested whether increased intestinal uracil availability alters susceptibility to rectal SIV infection in rhesus macaques. Despite limited evidence of rectal inflammation or epithelial damage, oral uracil treatment increased the number of viruses establishing infection, indicating increased susceptibility. Oral treatment also altered peripheral and small intestinal immune responses and perturbed the fecal microbiome. In contrast, uracil delivered directly to the large intestine by suppository increased markers of intestinal epithelial damage but did not increase SIV acquisition. These findings demonstrate that uracil can alter susceptibility to rectal SIV infection, but that increased susceptibility does not require overt damage to the rectal epithelial barrier. Our results highlight the potential for microbiome-associated metabolites to influence HIV transmission through mechanisms beyond local intestinal inflammation.

## Introduction

HIV is predominantly a sexually transmitted disease, with an estimated 1.3 million new infections occurring in 2024 [1]. Although widespread access to antiretrovirals and pre-exposure prophylaxes reduced global infection rates by 40% from 2010 to 2025 [1], vulnerable populations continue to experience high infection rates. Young women and MSM remain at particularly high-risk as compared to the broader population for reasons that are not fully understood. Despite the recent preventative successes achieved with the use of Lenacapavir and Tenofovir disoproxil fumarate-based therapeutics [2,3], reductions in global aid funding may exacerbate HIV infection rates [4]. The development of more cost-effective pre-exposure therapeutics and enhanced risk-based prevention guidelines necessitates a fuller understanding of inherent susceptibility to HIV and other sexually transmitted pathogens.

Known clinical risk factors for HIV ultimately increase susceptibility by inducing genital or gastrointestinal inflammation and immune activation [5,6]. Inflammation-associated apoptosis of epithelia compromises barrier integrity, activates target mucosal CD4+ T-cells, and recruits antigen presenting cells that first capture and then disseminate virions [7–9]. Several research groups have described microbial dysbiosis – the expansion of pathobionts relative to commensal taxa – as a correlate of HIV susceptibility [10–14]. Cervicovaginal *Lactobacillus* non-dominance is associated with an increased risk of HIV acquisition in women [10], and an expansion of penile anaerobes is associated with increased HIV seroconversion in men [13]. In contrast, a causal relationship between intestinal dysbiosis and susceptibility to intra-rectal HIV acquisition has been difficult to establish. Although the intestinal microbiome of MSM is characterized by enrichment of *Prevotella* and reduced abundance of *Bacteroides* [11,14], and MSM experience increased risk of HIV acquisition, it remains unclear whether these microbiome features directly contribute to increased susceptibility.

A balanced commensal microbiome is essential for calibrating health, including epithelial homeostasis. Commensal taxa metabolize essential dietary metabolites from consumed foods, program local and systemic immunity through antigenic and metabolic signaling, and compete with pathobionts for nutrients and anatomic niches [15]. Whereas short-chain fatty acid production by gastrointestinal Bacillota regulates epithelial stem-cell proliferation and differentiation, programs physiologic hypoxia, and initiates mucosal anti-microbial IL-22 production and T-regulatory helper cell differentiation, the loss of Bacillota by diet, disease, or antibiotic use permits aerobic pathobionts to bloom and to sustain localized inflammation [7]. In women, cervicovaginal *Lactobacillus* dominance benefits the individual through lactic acid production, which lowers pH to enhance epithelial barrier integrity and to inhibit colonization by bacterial and viral pathogens, including HIV [16]. Although retrospective analyses have identified microbiome-associated features that distinguish seroconversion from non-conversion in MSM exposed to HIV [12] or that discriminate between accelerated versus delayed acquisition in intra-rectally SIV-challenged non-human primates [17], these studies have not identified a mechanism associated with protection or lack thereof.

In rhesus macaques, we recently demonstrated that Vancomycin-mediated dysbiosis increases the risk of intra-rectal SIV acquisition [18]. Unexpectedly, we observed that perturbations to host anti-microbial immunity – rather than specific taxonomic changes – were predictive of increased susceptibility, suggesting that shared microbial antigens or metabolic outputs may contribute to increased risk of lentiviral infection. Among the identified indices of susceptibility were rectal IL-22 producing T-helper cell (T_H_22) frequencies and DUOX2 expression, two factors critical in the regulation of intestinal epithelial antimicrobial immunity*.* Whereas IL-22 regulates epithelial cell regeneration, mucous production, and antimicrobial peptide transcription [19], DUOX promotes epithelial ROS production to prevent the overgrowth of pathobionts in an uracil-dependent manner [20]. IL-22 further appears to regulate DUOX2 transcription [21].

The ubiquity of uracil is being exploited in the development of novel antibacterials, as a response to the emergence of antibiotic-resistant bacterial strains [22,23]. Evidence in the 1950s demonstrating that uracil is preferentially incorporated by some cancer cells *in vitro* spurred the widespread development of uracil derivatives and conjugates as chemotherapeutic and antimicrobial agents [24,25]. As nucleic acid analogs, these derivatives compete with natural nucleosides/nucleotides during RNA/DNA elongation leading to premature chain termination and/or mutagenesis. 5’- fluorouracil forms the basis for most currently used uracil-based chemotherapeutic pharmaceutics, with the prodrug Tegafur a standard of care for most gastrointestinal cancers [26]. Although the primary purpose of these uracil-based derivatives is to stall the replication of pathogens and cancerous cells, these drugs may also inadvertently increase susceptibility to heterologous infections and pathologies by altering host physiology and are deserving of wider scrutiny.

We hypothesized that bacterial dysbiosis may contribute to increased susceptibility to intra-rectal HIV/SIV by increasing the bioavailability of uracil at the gastrointestinal epithelial barrier. To experimentally address this, we provided both oral and suppository-compounded uracil to rhesus macaques prior to low-dose intra-rectal challenge with SIV. Oral uracil increased susceptibility to SIV infection and significantly perturbed the fecal microbiome without damaging gastrointestinal epithelial integrity. In contrast, uracil suppositories damaged the intestinal epithelial barrier without significantly altering the fecal microbiome or increasing susceptibility to SIV acquisition. Our results demonstrate that although exogenously provided uracil can both induce epithelial damage and increase susceptibility to intra-rectal SIV acquisition, uracil-enhanced susceptibility does not require rectal barrier damage. Further research is needed to identify the processes by which uracil may increase susceptibility to HIV infection and to determine whether these processes are susceptible to microbiome-labile therapeutics.

## Results

### Uracil treatment does not efficiently induce epithelial cell ROS production *in vitro*

To model the effects of uracil on the primate intestinal epithelium, we first assessed the effects of uracil on Caco-2 cell monolayers *in vitro*. We cultured Caco-2 cells in aerobic conditions on tissue cultured 6-well plates for 3 days to achieve confluency and assessed whether uracil was sufficient to induce ROS after 24 hours of stimulation. Whereas supernatant from Caco-2 cells incubated with ROS inducer Tert-butyl hydroperoxide (TBHP) showed increased fluorescence of the ROS probe 2’,7’- dichlorofluorescein (DCF) as compared to vehicle-only (DMSO) Caco-2 cell controls, DCF median fluorescence was comparable between controls and uracil-treated Caco-2 cells (S1A Fig). We further measured proliferation by Ki67 expression and cell death by viability (amine) staining by flow cytometry - neither uracil- nor TBHP-treated Caco-2 cells differed significantly from controls (S1B and S1C Fig).

To better approximate *in vivo* conditions, we next differentiated Caco-2 cells in aerobic conditions on Transwell inserts with exposure to uracil, uridine, and TBHP beginning 3 days after seeding and continuing out to day 17 post-seeding. Supernatant collected from transwell-cultured Caco-2 cells with continuous TBHP and uridine exposure showed significantly increased apical ROS production as compared to supernatants from vehicle-exposed cells - apical ROS production from uracil-exposed cells did not differ from controls (S1D Fig). In these transwell conditions, neither uracil nor uridine altered Caco-2 cellular proliferation, viability, nor the development of monolayer integrity as measured by transepithelial electrical resistance (TEER), as compared to the vehicle-only controls (S1E–S1G Fig). Our results suggest that Caco-2 intestinal monolayers may be insufficient for fully modeling the dynamic intestinal epithelial interface.

### Oral uracil treatment does not induce intestinal inflammation

To identify whether uracil induces intestinal inflammation and increases susceptibility to intra-rectal SIV acquisition, we designed a longitudinal, *in vivo* study incorporating both pre- and post-challenge assessments (Fig 1A). During the pre-challenge phase, we provided rhesus macaques (*Macaca mulatta*) with 125 mg/m^2^ oral uracil, twice daily, with food until animals became SIV-infected (up to 7 months). The dose used was estimated on previously described Tegafur dosing in adult humans [26]. We collected blood and stool every 1–2 weeks during this phase and acquired intestinal biopsies at baseline (Pre) and 4 weeks into treatment (Post). Macaques received uracil for an additional 4 weeks and we continued to collect blood and stool - no additional biopsies were collected to allow for surgical recovery prior to challenge. Eight weeks after treatment initiation, macaques underwent bi-weekly low-dose intra-rectal challenges with 4 TCID50 SIVmac239X [27]. Plasma was assessed for viral RNA at days 7 and 14 post-challenge. Uracil treatment, blood and stool collections, and challenges continued until infection was confirmed at which point, treatment and challenges stopped.

**Fig 1:**
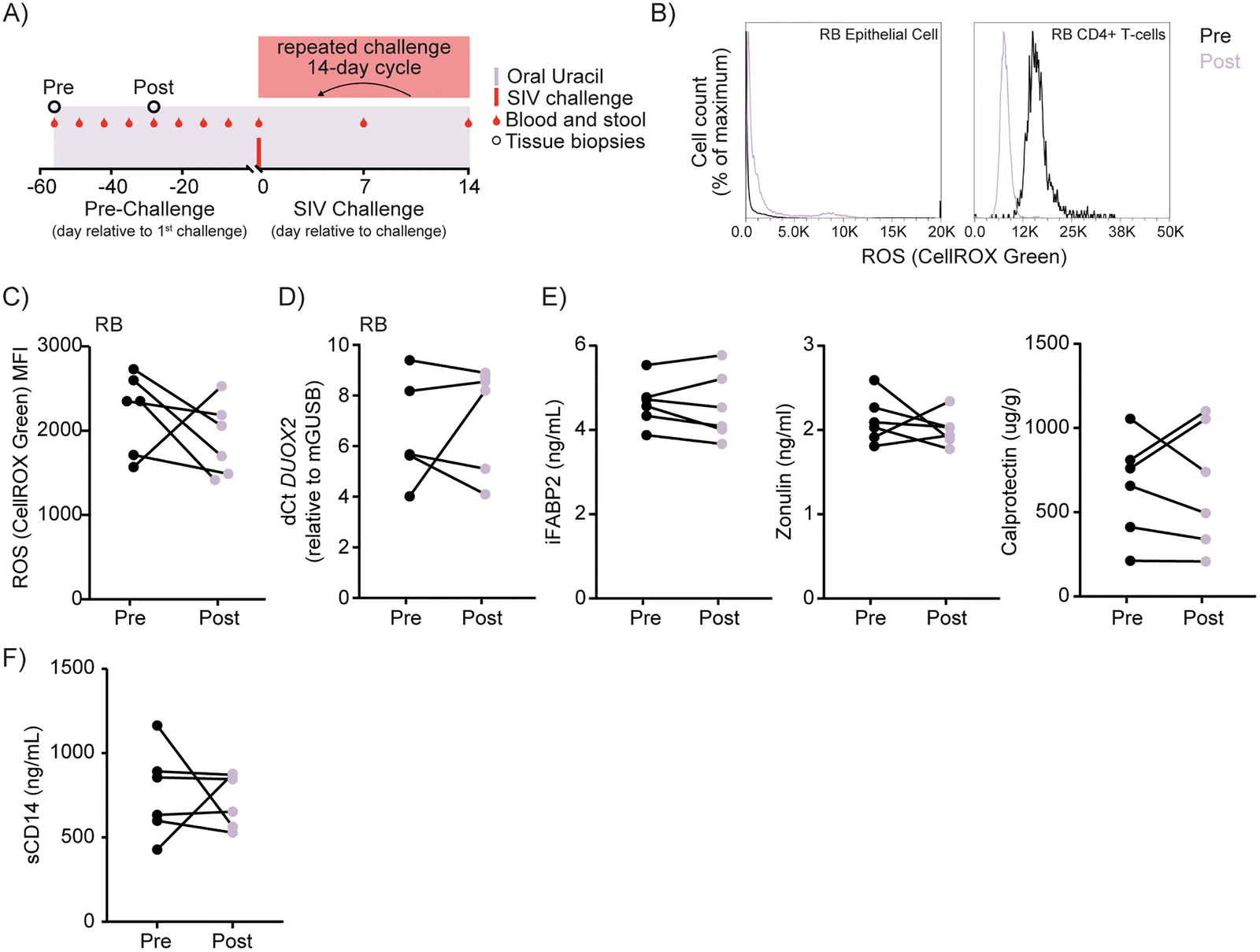
Oral uracil treatment does not damage the intestinal epithelial barrier. (A) Study design. Our study was composed of a pre-challenge phase where responses to oral uracil treatment were established, and a challenge phase during which animals underwent low-dose intra-rectal challenges with SIVmac239X. Within the pre-challenge phase, both Pre-treatment (Pre) and Post-treatment (Post) samples were collected as indicated. (B) Representative flow cytometry histograms depicting ROS (CellROX Green) fluorescence in RB epithelial and CD4+ T-cells. (C) ROS MFI in RB epithelial cells. (D) Relative expression of *DUOX2* in RB homogenates. (E) Indirect markers of intestinal damage plasma iFABP2 and zonulin, and fecal calprotectin, as assessed by ELISA. (F) Indirect marker of microbial translocation, plasma sCD14 as assessed by ELISA. Significance in C-F assessed by paired, one-way t-test.

Both ingested and microbiome-derived uracil have been shown to increase epithelial-associated ROS in *Drosophila* models in a DUOX-dependent manner [20]. To assess whether oral uracil treatment increases epithelial ROS production in rhesus macaques, we identified epithelial cells by flow cytometry (S2 Fig) from rectal single-cell suspensions and stained for ROS *ex vivo* using the fluorogenic probe CellROX Green (Fig 1B). No significant increase in ROS median fluorescence intensity (MFI) was observed in a longitudinal comparison of rectal epithelial cells (Fig 1C). We additionally measured *DUOX2* expression by quantitative reverse-transcription PCR (qRT-PCR) in bulk rectal tissue homogenates and observed no significant increase (Fig 1D).

Homeostatic and acute inflammation at the gastrointestinal barrier accelerate epithelial shedding and recruit inflammatory immune cells for the purposes of limiting pathogen colonization and invasion; however, prolonged inflammation can diminish barrier integrity and lead to increased pathogen susceptibility and microbial translocation [7]. To assess whether prolonged oral uracil treatment compromises barrier integrity, we measured indirect markers of gastrointestinal integrity - plasma intestinal fatty acid binding protein 2 (iFABP2) and zonulin, and fecal calprotectin - by ELISA [28]. We did not observe increases in these markers post-treatment, suggesting that orally-provided uracil does not significantly weaken gastrointestinal barrier integrity (Fig 1E). We lastly considered that oral uracil treatment might induce microbial translocation, irrespective of significant barrier damage. To assess this potential, we quantified soluble CD14 (sCD14) in plasma but did not observe a longitudinal increase (Fig 1F). Our results suggest that orally-provided uracil treatment does not perturb rectal inflammation or weaken intestinal barrier integrity.

### Oral uracil treatment perturbs non-rectal immunity

The intestinal oxidative stress response, microbiome, and DUOX2 transcription are associated with IL-22 regulation at the epithelial barrier by mechanisms that are not fully understood [20,21] but which can be manipulated by pathogenic taxa [29]. To determine whether oral uracil treatment disrupted intestinal IL-22 production, we stimulated peripheral blood mononuclear cells (PBMCs) and mononuclear cell suspensions isolated from rectal (RB) and jejunal (Jej) biopsies with phorbol 12-myristate 13-acetate (PMA)-ionomycin and performed intracellular cytokine staining. As assessed within gated T-cells by flow cytometry, IL-22 production by intestinal CD4+ memory T-cells was not significantly perturbed; however, we observed significantly decreased IL-22 production among CD4+ TM PBMCs (Fig 2A; PBMC *p = 0.0044*). We observed the same pattern when we extended our analysis to include IL-17, IFNγ, and TNFα production and proliferation as measured by Ki67. Surprisingly, whereas oral uracil treatment did not alter the intestinal CD4+ memory T-cell (TM) profiles we measured, treatment significantly decreased cytokine expression and proliferation among CD4+ memory T-cell (CD4+ TM) PBMCs (Fig 2B). A similar but muted pattern was observed for CD8 + TM, where IL-22 and IFNγ production were decreased post-treatment in PBMCs – no differences were seen for the remaining examined markers or tissue sites (S3A Fig).

**Fig 2:**
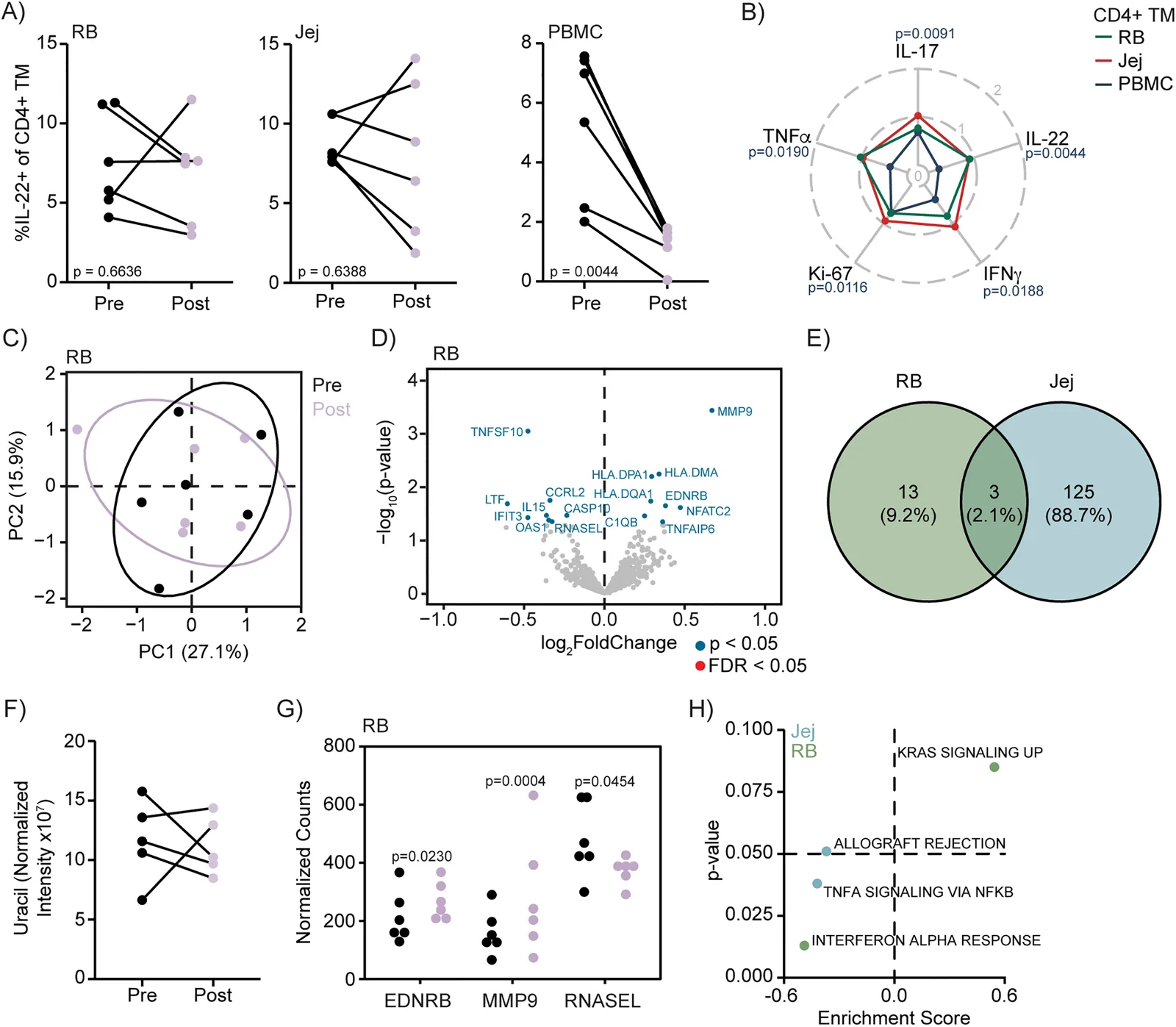
Oral uracil treatment perturbs immunity at non-rectal sites. (A) Percent IL-22 expression of RB, Jej, and PBMC CD4+ TM Pre- and Post-oral uracil treatment. (B) Radar plot depicting fold-change from Pre-treatment expression of markers/cytokines in CD4+ TM at indicated sites in oral uracil-treated macaques. Cytokine expression measured in response to *ex vivo* PMA/ionomycin stimulation. p-values evaluate the difference of expression values at the Pre versus Post timepoints within each tissue and are color-coded by tissue site. (C) PCA considering NanoString-enumerated RB transcripts from oral uracil-treated macaques Pre- and Post-treatment. (D) Volcano plot depicting the fold-change expression of NanoString-enumerated RB transcripts at the Post-treatment timepoint. Transcripts are color-coded by significance. (E) Venn diagram depicting the number of NanoString-enumerated transcripts identified as differentially expressed within RB and Jej homogenates. (F) Normalized intensity of fecal uracil as identified by untargeted LC-MS. (G) Normalized counts of RB gene transcripts identified as differentially expressed within both RB and Jej homogenates. (H) Pathways associated with differentially abundant transcripts among RB and Jej homogenate transcripts. Pathways are color-coded by tissue site. Lines in B denote per-site, fold-change from Pre-treatment means. Significance in A assessed by paired two-way t-test, in B by paired two-way t-test on frequency-of-CD4+ TM expression values, in C by adonis (PERMANOVA), in D-E and G by DESeq2, in F by paired one-way t-test, and in H by GSEA.

To examine whether oral uracil treatment more broadly perturbed intestinal immunity, we next assessed intestinal transcriptomics by NanoString. Considering 769 immunologic transcripts, we observed that oral uracil treatment did not broadly perturb rectal immunity as visualized by principal component analysis (PCA) (Fig 2C). As assessed by DESeq2, 16 rectal genes were differentially expressed following oral uracil treatment (p < 0.05), though none survived FDR correction (Fig 2D). We did, however, observe a trend towards disrupted jejunal immunity (S3B Fig). One hundred twenty-eight genes exhibited differential expression among jejunal homogenates, including 49 passing FDR – these included an upregulation of *IFNAR2, ATG5,* and *DDX50* and a downregulation of *CD79B, BCL6*, and *CTNNB1* (S3C Fig). Given the extent of jejunal transcript perturbations, we assessed whether epithelial ROS levels were perturbed in jejunal epithelial cells and unexpectedly, we observed significantly decreased ROS production post-treatment (S3D Fig), indicating that both immunity and inflammation were perturbed within the small intestine. Thus, we considered that uracil might be absorbed or metabolized within the small intestine, thereby limiting bioavailability within the large intestine lumen. Indeed, we did not observe significant longitudinal alterations in fecal uracil by untargeted metabolomics, suggesting that ingested uracil did not reach the large intestine in appreciable quantities (Fig 2F).

Although oral uracil treatment did not overwhelmingly perturb rectal inflammation or immunity, isolated perturbations might contribute to rectal epithelial barrier dysfunction. To narrow down uracil-responsive genes within the rectal environment, we identified 3 gene/gene families that were shared between our rectal and jejunal transcript analyses. At both tissue sites, *EDNRB* and *MMP9* were upregulated following oral uracil treatment, and *RNASEL* was downregulated (Figs 2G and S3E). Notably, *MMP9* and *EDNRB* contribute to extracellular matrix and endothelial remodeling, respectively [30,31], suggesting that oral uracil treatment may potentiate intestinal tissue remodeling. Pathway analyses of transcripts quantified in these tissues identified the “Interferon alpha response” pathway and the “TNFα signaling via NFKb” pathways as decreased in rectal and jejunal tissues, respectively, post-treatment (Fig 2H). We further observed trends for enriched “KRAS signaling up” pathway activity in rectal tissues and decreased “Allograft rejection” pathway activity in jejunal tissues. These data demonstrate that oral uracil treatment induced perturbations to peripheral blood and jejunal immunity and modest changes to immunity and inflammation within the large intestine.

### The fecal microbiome is responsive to oral uracil treatment

In *Drosophila*, non-commensal, bacterial-derived uracil induces intestinal ROS production in a DUOX-dependent manner which, when properly regulated, leads to pathobiont control [20,32]. As ROS-mediated pathobiont control is beneficial to the host, it follows that sustained uracil production by pathobionts likely confers a competitive advantage against commensal competitors. To determine if oral uracil treatment perturbs the primate intestinal microbiome, we measured the composition of the fecal bacterial microbiome by 16S Illumina sequencing and assessed differential abundance by ANCOM-BC. Broadly, the distribution of bacterial phyla did not differ from Pre- to Post-treatment or to the time of the first challenge (Chall; Fig 3A). However, the relative abundance of the Thermodesulfobacteriota phylum significantly increased from Pre-treatment to Challenge (Fig 3B, p = 0.0423). To determine if abundance differed at lower taxonomies, we further assessed both family and amplicon sequence variant (ASV) abundance. We observed family-level perturbations across many phyla, with no clearly unifying pattern (Fig 3C). Among Pseudomonadota, *Moraxellaceae* and *Xanthomonadaceae* were diminished and *Sutterellaceae* increased at the Challenge timepoint relative to Pre-treatment. Several changes were also observed within the Bacillota phylum, which had diminished *Vagococcaceae* at challenge and enriched *Butyricicoccaceae*, *Erysipelatoclostridiaceae*, and *Selenomonadaceae*. Within the Bacteroidota phylum we observed decreased *Weeksellaceae* and increased *Prevotellaceae* and *Rikenellaceae* at challenge. Unexpectedly, modest perturbations at the phylum and family levels masked marked shifts among ASVs, which exhibited decreased relative abundance overall. One-hundred-ten ASVs were significantly altered between Pre-treatment and Challenge and were again concentrated among Bacteroidota, Bacillota, and Pseudomonadota (Fig 3D).

**Fig 3:**
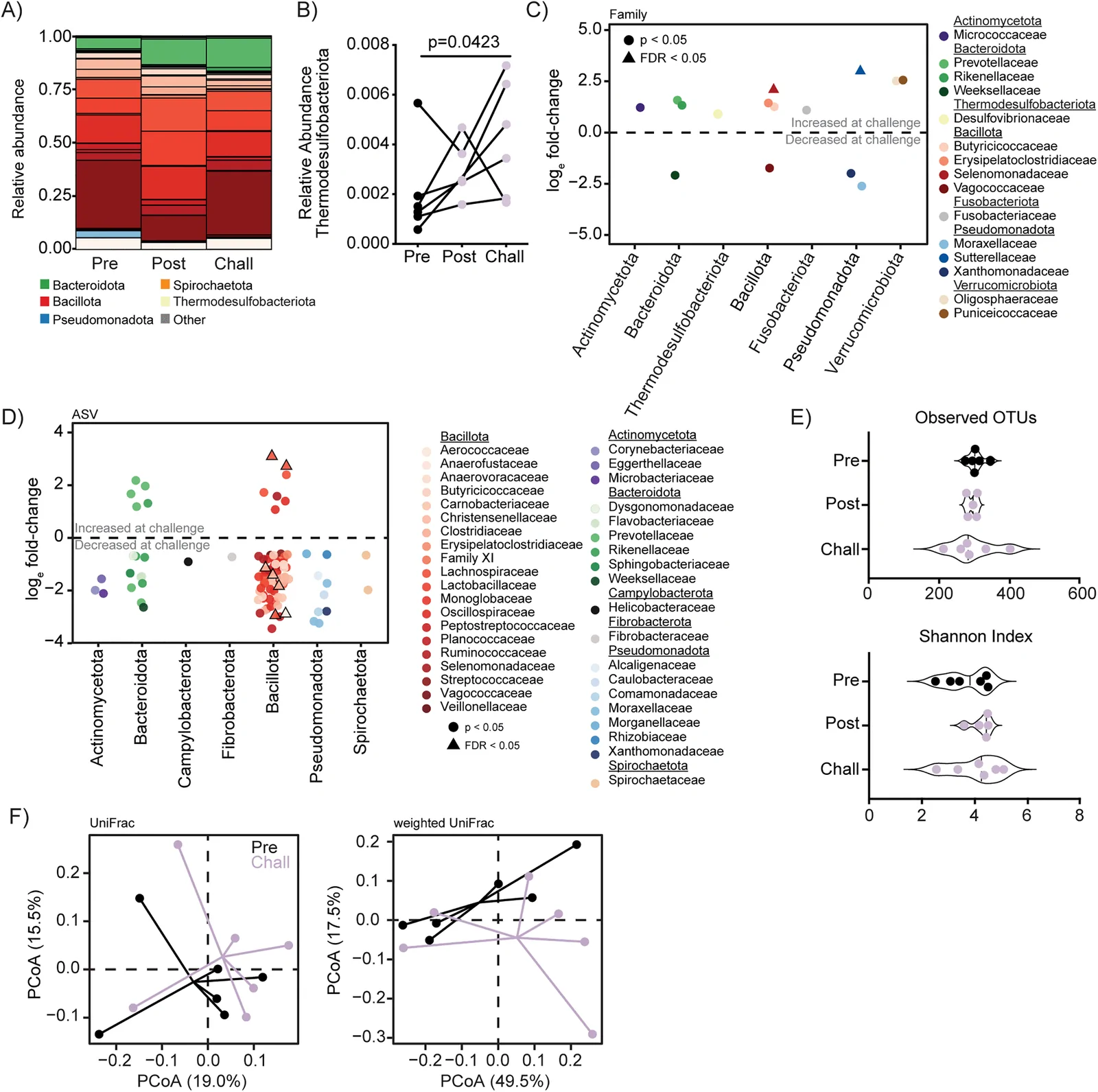
Oral uracil treatment perturbs the fecal microbiome. (A) Relative fecal bacterial abundance in oral uracil-treated animals at Pre-treatment, Post-treatment, and on the day of the first SIV challenge (Chall). Taxa are color-grouped by phylum and individually shaded by family as in C. (B) Relative fecal bacterial abundance of Thermodesulfobacteriota in oral uracil-treated animals across the pre-challenge phase. (C-D) Fold-change frequencies of fecal families (C) and ASVs (D) identified as longitudinally, differentially abundant at the time of challenge relative to Pre-treatment. Data show the natural log fold-change, color-grouped by phylum and individually shaded by family or ASV. (E) Richness (Observed OTUs) and Shannon indices of fecal bacterial α-diversity across the pre-infection period. (F) PCoA of unweighted and weighted UniFrac measures of fecal bacterial β-diversity. Bar segments in A denote mean relative abundance. Lines in E denote mean α-diversity. Significance in A-B and E calculated by paired two-way t-test, in C-D by ANCOM-BC, and in F by adonis (PERMANOVA).

Alterations to keystone species within the intestinal microbiome can have widespread effects on microbial community structure [33], without perturbing higher-level taxa representation. To determine if differences in ASV and family abundance extended to community restructuring, we measured α- and β- diversity across the pre-challenge phase. No differences were seen for richness (Observed OTUs) or Shannon indices of α-diversity (Fig 3E), nor were differences observed for unweighted and weighted UniFrac measures of β-diversity as assessed by principal coordinate analysis (PCoA) (Fig 3F), suggesting that oral uracil treatment perturbed fecal bacterial families and ASVs without globally restructuring the fecal microbiome.

### Oral uracil treatment increases susceptibility to SIV acquisition

Although oral uracil treatment did not significantly alter fecal uracil, rectal immunity, or epithelial integrity, alterations to jejunal immunity and the fecal microbiome confirmed that intestinal changes were induced. To determine if oral uracil treatment altered susceptibility to intra-rectal SIV acquisition, we repeatedly challenged our cohort with a low-dose of SIVmac239X, which allowed for quantification of both the number of challenges required for successful infection as well as the number of acquired transmitted-founder (T/F) variants [27]. As compared to contemporaneously challenged macaques, we observed no significant alteration in the number of challenges required for successful SIV acquisition (Fig 4A). We did, however, observe a significant increase in the number of T/F variants acquired by oral uracil treated animals as compared to contemporaneous controls, suggesting that treatment increased susceptibility to intra-rectal SIV acquisition (Fig 4B). We did not observe significant correlations between the number of T/F variants acquired and the Post-treatment fecal uracil concentration, *DUOX2* dCt, ROS MFI, and IL-22 expression in CD4+ TM. Increased acquisition by oral uracil-treated animals was not associated with altered acute plasma viral kinetics as compared to controls, with both groups showing comparable peak and early post-peak plasma viral loads (Fig 4C).

**Fig 4:**
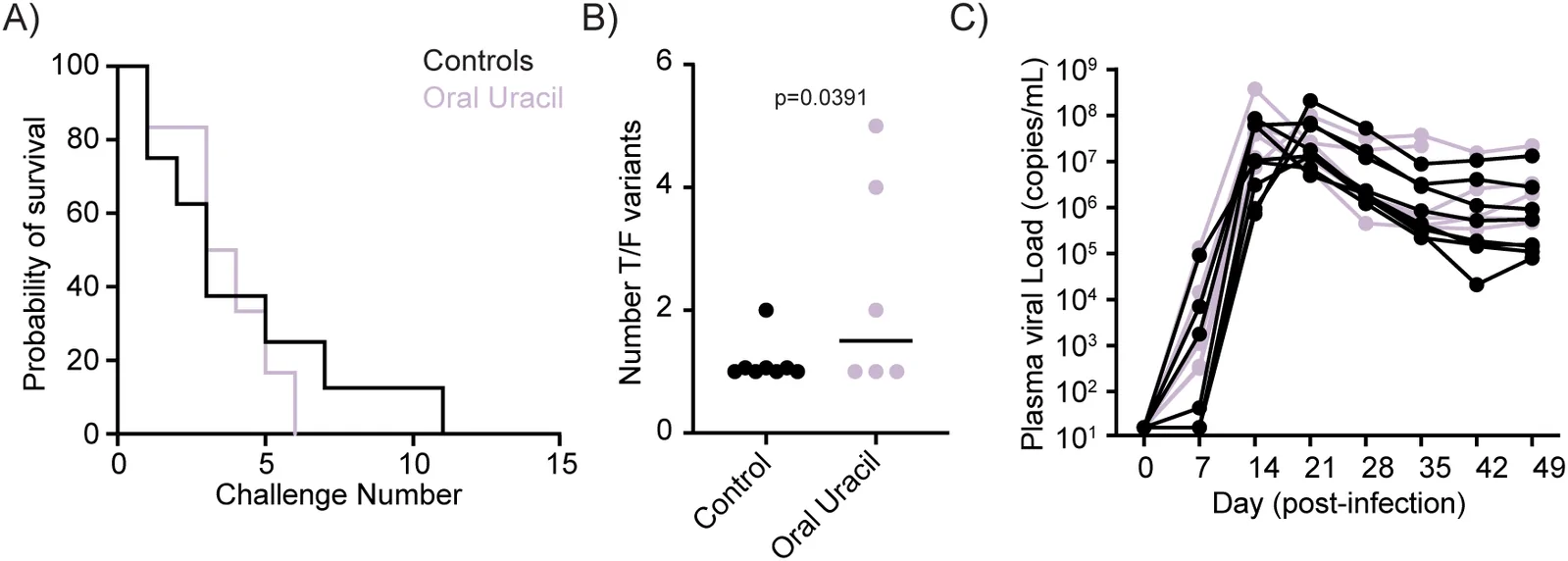
Oral uracil treatment increases the number of T/F variants following intra-rectal SIV challenge. (A) Survival curve depicting the remaining percent of uninfected animals after each challenge. Control animals include independently assayed, chronologically matched challenge controls. (B) Number of T/F variants detected in acute SIV infection. (C) Longitudinal SIV plasma viral load during acute SIV infection. Lines in A-B represent means. Significance in A calculated by Log-rank (Mantel-Cox) test, in B by one-way, unpaired t-test, and in C by mixed-effects model (REML).

### Uracil suppositories induce modest intestinal inflammation

Although oral uracil treatment increased the susceptibility of rhesus macaques to intra-rectal SIV acquisition, it did so independent of detected alterations to the rectal epithelium, likely resulting from poor luminal bioavailability. To ascertain whether uracil availability specifically within the large intestine contributes to increased susceptibility, we repeated our study, instead using uracil suppositories. Here, animals received thrice-weekly suppositories of uracil compounded with cocoa butter, with the sampling and challenge schedule identical to that used in the oral treatment cohort (Fig 5A). Cocoa butter was chosen because it is listed as an inactive, suppository compounding reagent by the FDA, has few reported interactions with the intestinal microbiome, and has the appropriate viscosity at body temperature for the prolonged retention of pharmaceuticals [34,35]. Treatment with uracil suppositories was associated with a trend for increased RB epithelial ROS expression (Fig 5B), but no alteration to longitudinal rectal homogenate *DUOX2* transcript levels (Fig 5C). In contrast to oral uracil treatment which induced a significant decrease in jejunal ROS (S3D Fig), uracil suppositories induced a significant increase in jejunal epithelial ROS (S4A Fig).

**Fig 5:**
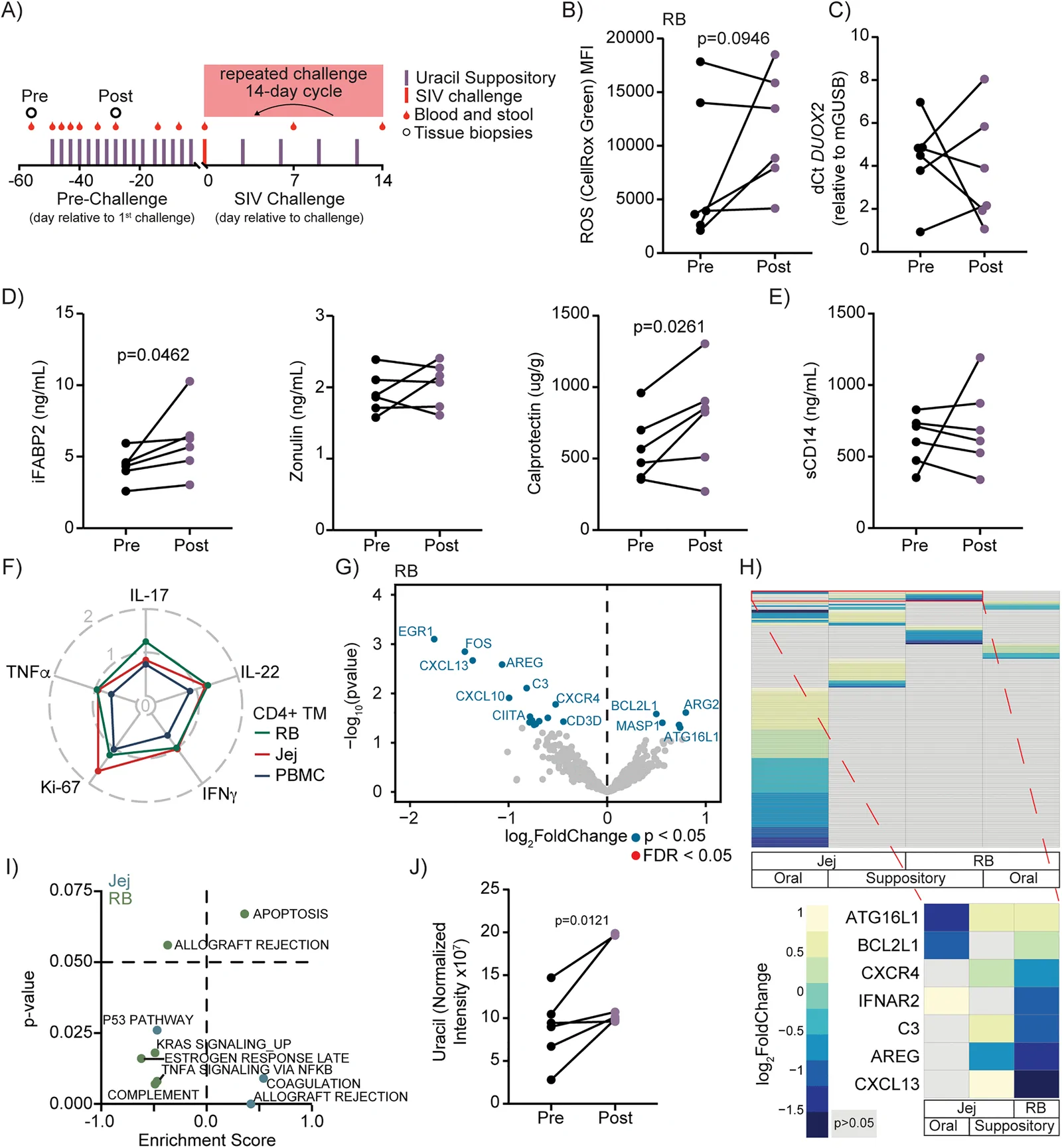
Uracil suppositories damage the intestinal epithelium while minimally perturbing systemic immunity. (A) Study design for uracil suppository treatment. Sample collections as in Fig 1A. (B) ROS MFI in RB epithelial cells. (C) Relative expression of *DUOX2* in RB homogenates. (D) Indirect markers of intestinal damage plasma iFABP2 and zonulin, and fecal calprotectin, as assessed by ELISA. (E) Indirect marker of microbial translocation, plasma sCD14 as assessed by ELISA. (F) Radar plot depicting fold-change from Pre-treatment expression of markers/cytokines in CD4+ TM at indicated sites in uracil suppository-treated macaques. Cytokine expression measured in response to *ex vivo* PMA/ionomycin stimulation. (G) Volcano plot depicting the fold-change expression of NanoString-enumerated RB transcripts at the Post-treatment timepoint. Transcripts are color-coded by significance. (H) Heatmap depicting fold-change expression of intestinal transcripts (rows) identified to be longitudinally, differentially abundant for the site/treatment shown. Inset highlights transcripts that were differentially abundant following rectal suppository and one additional site or condition. Transcripts are manually sorted by site, treatment, fold-change, and overlap to emphasize shared transcripts. (I) Pathways associated with differentially abundant transcripts among RB and Jej homogenate transcripts. Pathways are color-coded by tissue site. (J) Normalized intensity of fecal uracil as identified by untargeted LC-MS. Lines in F denote per-site, fold-change from Pre-treatment means. Significance in B-E and J assessed by paired one-way t-test, in F by paired two-way t-test on frequency-of-CD4+ TM expression values, in G-H by DESeq2, and in I by GSEA.

To further examine whether uracil suppositories increased intestinal permeability, we quantified plasma iFABP2 and zonulin, and fecal calprotectin by ELISA. Here, we observed significant longitudinal increases in iFABP2 and calprotectin (Fig 5D). No increases were observed in plasma sCD14 (Fig 5E), suggesting that epithelial damage did not induce microbial translocation or systemic inflammation. Indeed, no significant longitudinal differences were observed for expression of IL-17, IL-22, Ki67, or TNFα by either CD4+ TM (Fig 5F) or CD8 + TM (S4B Fig). A significant decline in IFNγ expression was observed from among peripheral blood CD8 + TM only, indicating that uracil suppositories did not widely alter intestinal or peripheral blood immune function.

To determine whether uracil suppositories altered intestinal transcriptomics, we assessed rectal and jejunal intestinal homogenates by NanoString. We did not observe significant differences by PCA for either tissue site (S4C Fig). Twenty genes were differentially expressed in rectal tissues and 39 in jejunal tissues, with none passing FDR (Figs 5G and S4D). We next identified shared differentially expressed genes across rectal and jejunal homogenates and both methods of uracil delivery. Seven genes were identified as differentially regulated in rectal homogenates from suppository-treated animals and in jejunal homogenates following either oral or suppository treatment (Fig 5H). Surprisingly, there were no shared genes identified when comparing rectal tissues between oral and suppository uracil treatment. Five genes were shared in both the rectal and jejunal analyses from suppository-treated animals – *AREG, ATG16L1, C3, CXCL13,* and *CXCR4* – with only *AREG* (decreased) and *ATG16L1* (increased) sharing directionality. Transcripts for ATG16L1 – a mediator of Paneth cell homeostatic autophagy [36] – were further identified as significantly decreased in jejunal homogenates from orally-treated animals. *BCL2L1* and *IFNAR2* were differentially regulated in jejunal homogenates from orally treated macaques and in rectal homogenates from suppository-treated macaques; however, the effects were opposing by tissue site.

We next characterized the specific pathways that were altered by uracil suppository treatment and observed a greater degree of disruption as compared to oral treatment. Pathway analysis of rectal transcripts demonstrated that the “Complement”, “TNFα Signaling via NFκB”, “Estrogen Response Late”, and “KRAS Signaling Up” pathways showed significantly decreased enrichment whereas “Allograft Rejection” (decreased) and “Apoptosis” (increased) pathways displayed trends for altered regulation (Fig 5I). Analysis of jejunal transcripts showed significantly increased enrichment of the “Coagulation” and “Allograft Rejection” pathways, and decreased enrichment of the “P53 pathway”. By untargeted metabolomics, we further observed increased fecal uracil in suppository-treated macaques, verifying the bioavailability of uracil within the intestinal lumen (Fig 5J).

### Uracil suppositories do not significantly alter the intestinal microbiome

Given the alterations observed in intestinal epithelial ROS levels, barrier integrity, and fecal uracil bioavailability following oral treatment, we expected to see even greater alterations of the intestinal microbiome following suppository treatment. Although a significant increase in Bacillota was observed from Pre- to Post-treatment (p = 0.0378), this did not persist to the time of challenge and no other differences were seen at the phylum level (S5A Fig). Modest longitudinal differences were noted among ASVs (S5B Fig) - ASV perturbations were seen predominantly among Bacillota from Pre-treatment to Challenge, but without the biased directionality seen following oral treatment. Reflecting the limited changes in phylum and ASV abundance, uracil suppository treatment was not associated with significant differences in either richness or Shannon α-diversity, or in unweighted or weighted UniFrac β-diversity as assessed by PCoA (S5C and S5D Fig).

### Uracil suppositories do not increase susceptibility to SIV acquisition

Finally, although we observed increased intestinal permeability in response to uracil suppositories, we did not observe significant differences in susceptibility to low-dose intra-rectal challenge with SIVmac239X as compared to contemporaneous controls. No differences were observed in either the number of challenges required for infection or the number of T/F variants acquired (Fig 6A and 6B). Furthermore, no differences were observed in acute viral dynamics (Fig 6C). These results suggest that as formulated for this study, uracil suppositories do not increase susceptibility to intra-rectal SIV acquisition.

**Fig 6:**
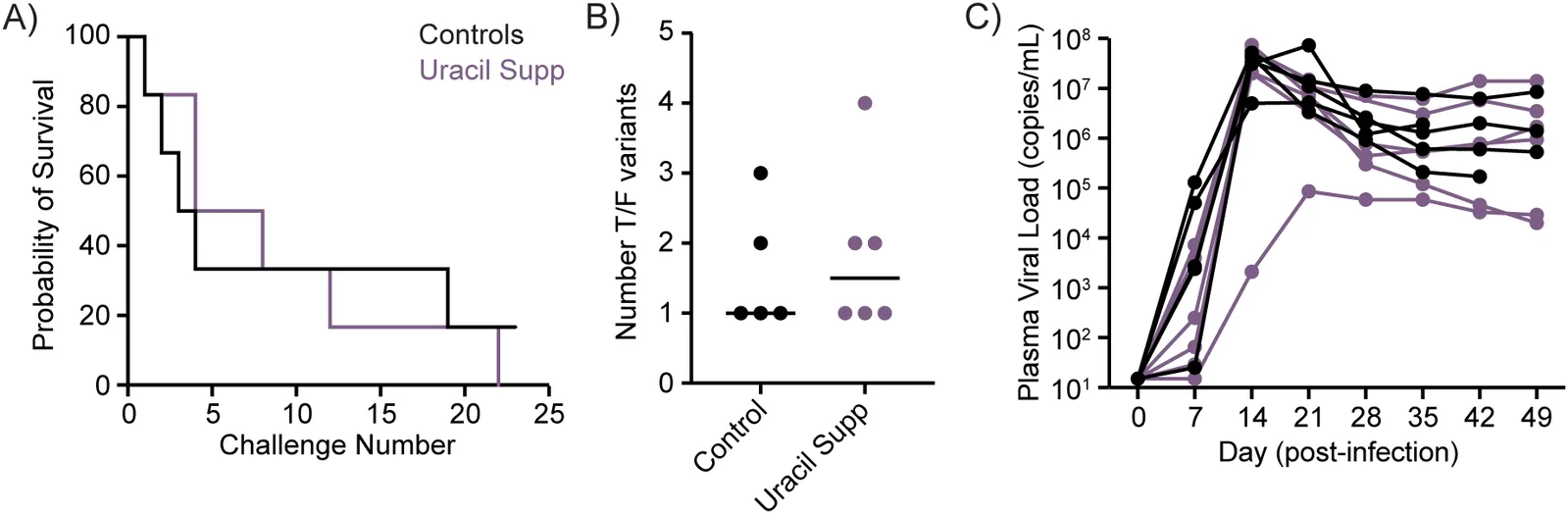
Uracil suppositories do not increase susceptibility to intra-rectal SIV acquisition. (A) Survival curve depicting the remaining percent of uninfected animals after each challenge. Control animals include independently assayed, chronologically matched challenge controls. (B) Number of T/F variants detected in acute SIV infection. (C) Longitudinal SIV plasma viral load during acute SIV infection. Lines in A-B represent means. Significance in A calculated by Log-rank (Mantel-Cox) test, in B by one-way, unpaired t-test, and in C by mixed-effects model (REML).

## Discussion

Understanding how the microbiome contributes to unique susceptibilities to HIV in highly at-risk populations is a critical benchmark for the development of improved pre-exposure prophylaxes. Although clear associations have been shown between specific vaginal and penile bacterial communities and susceptibility to HIV acquisition at these sites [10,13], no clear association has been found between the gastrointestinal microbiome and rectal acquisition. Using a rhesus macaque model, we recently observed that vancomycin-mediated dysbiosis led to alterations in rectal T_H_22 frequencies and DUOX2 transcript abundance, and in parallel, an increased risk of intra-rectal SIV acquisition [18]. As other reports have demonstrated that IL-22 and microbiota-derived uracil diametrically regulate DUOX2-mediated intestinal epithelial inflammation [19–21], we hypothesized that uracil derived by a dysbiotic intestinal microbiome may contribute to susceptibility to intra-rectal HIV acquisition. Herein, we demonstrate that exogenously provided uracil can increase susceptibility to intra-rectal SIV acquisition independent of epithelial inflammation.

Uracil has been studied extensively for its direct role in limiting lentiviral replication. The cytidine deaminase and host restriction factor Apolipoprotein B mRNA Editing Enzyme Catalytic Subunit 3G (APOBEC3G) induces excessive viral DNA uracilation to interfere with viral replication and in turn, is antagonized by viral protein Vif in a species-specific manner [37]. Deamination by APOBEC3G is not thought to influence the early stages of viral replication but rather, heavily uracilated HIV and SIV DNA fail to integrate into the host genome, in part due to elongation inhibition [37,38]. Whereas excessive mucosal uracil potentiates viral hyper-uracilation and abortive infection of target CD4+ T-cells within intestinal tissues, luminal uracil may modulate susceptibility to viral transmission across the intestinal epithelial barrier. Indeed, we observed significantly increased seeding by transmitter/founder variants in our oral uracil-treated macaques. However, this effect was modest and the group-level difference was influenced by a small number of animals with high T/F variant counts, while the number of challenges required for infection was unchanged. Furthermore, in this small cohort, we did not identify correlations between T/F variant number and fecal uracil concentration, or RB *DUOX2* expression, epithelial ROS, or IL-22 expression in CD4+ TM, limiting our ability to identify a specific correlate of increased T/F acquisition. Curiously though, in uracil suppository-treated macaques where intestinal epithelial damage was evident and fecal uracil was significantly elevated, we did not observe a significant difference in susceptibility to infection as measured by either number of challenges required for successful infection or number of T/F variants acquired. The mechanism(s) underlying this discordance are unclear. Epithelial damage is itself a consistent correlate of mucosal inflammation and of HIV and SIV susceptibility [8,39]. In part, our results could be due to the use of cocoa butter as the suppository vehicle. Either the biochemical or biophysical properties of cocoa butter may have interfered with luminal SIV transmissibility; however, we were unable to address these variables in the present study.

Because of uracil’s well-documented role in precipitating DNA damage when in excess, uracil derivatives have been studied extensively for use as chemotherapeutic and antiviral agents, including against HIV [25]. Clinical studies reporting on the safety and mechanisms of action of these derivatives offer some insight into our outcomes. We observed that orally provided uracil alters jejunal immunity to a greater extent than rectal immunity without increased fecal uracil output. Orally administered uracil and 5’-fluorouracil are readily absorbed through the small intestine, prior to first-pass metabolism within the liver [40,41]. Absorption and luminal bioavailability of pyrimidines across the large intestine, however, are variable for reasons that remain poorly understood [42,43]. Although this anatomic disparity can in part be attributed to the restricted expression of the sodium-dependent nucleobase transport 1 (SNBT1) in non-primate mammals, the mechanisms concerning uracil transport in primates are unclear with bioavailability of 5’-fluorouracil widely variable after both oral and rectal treatment [42,44]. Despite observing significantly increased fecal uracil following suppository treatment, we still observed greater transcriptional changes in jejunal than rectal tissues following suppository-provided uracil, and observed that many differentially regulated jejunal transcripts were shared across treatment type (Fig 5H). These results may reflect the absorption, metabolism, and redistribution of luminal contents through hepatic circulation [45].

Route-dependent differences in exposure may contribute to the markedly distinct rectal transcriptional responses observed following oral and suppository uracil treatment in our study. Oral administration is likely to favor proximal intestinal absorption followed by metabolism and redistribution, whereas suppository administration is likely to result in greater measurable luminal uracil availability within the distal gastrointestinal tract. Consequently, rectal tissues may have been exposed to different concentrations or durations of uracil, uracil-derived metabolites, or systemic signals depending on the route of administration. The distinct dosing schedule and localized delivery of suppositories also differed substantially from twice-daily oral administration, and an independent effect of the cocoa butter vehicle on the local mucosal environment cannot be excluded. Thus, the absence of shared differentially expressed genes in rectal tissues between the two treatment routes may reflect differences in pharmacokinetics, tissue distribution, and mucosal exposure. Retrospective studies linking metabolite availability to HIV seroconversion such as that by Fulcher et al. [12] and methodical *in vitro* mixed bacterial/intestinal co-cultures such as those by Dillon et al. [46] are needed to gain further insight into the complex metabolic dynamics that govern HIV susceptibility.

The composition of the intestinal microbiome likely contributes to variability of intestinal uracil absorption in outbred primate populations. Antibiotics alter the efficacy of 5’-fluorouracil by both direct and indirect mechanisms [47–49] and uracil transport may be inhibited under anaerobic conditions [43] such as that maintained by commensal taxa at the colonic epithelium [20,50]. In turn, uracil derivatives can alter the composition of the microbiome. Experimental murine models of 5’-fluorouracil-induced intestinal mucositis have reported decreased intestinal and fecal abundance of Bacillota [51,52]. In our oral uracil cohort, we observed significant declines in many Bacillota, Pseudomonadota, and Bacteroidota ASVs independent of alterations to measures of bacterial diversity. Among Bacillota, decreased ASVs in the oral cohort were heavily skewed towards Clostridia (59/71). The loss of Clostridia was not associated with increased rectal ROS or epithelial damage in our oral uracil model, where in contrast, we observed intestinal damage without significant microbiome perturbations in our suppository model. These results may suggest that hepatic uracil catabolism and by extension the availability of uracil catabolites, has a greater effect on the composition of the microbiome than the luminal bioavailability of uracil itself [41,53]. Indeed, amino acid and secondary bile acid metabolism have previously been identified as key metabolic features associated with increased HIV acquisition risk [12] and disease progression [54] in PLWH.

Intriguingly, no longitudinal alterations were observed in rectal *DUOX2* transcription for either treatment, suggesting that exogenously administered uracil is not sufficient to alter rectal *DUOX2* transcription in the primate model. These results contrast with previous observations reported using *Drosophila melanogaster* [20] and highlight the additional complexity associated with modeling the contribution of the microbiome to intestinal inflammation in non-human and human primates. Indeed, we were unable to model intestinal inflammation using a Caco-2 model *in vitro* (S1 Fig)*.*

Here, we demonstrated that uracil can increase susceptibility to intra-rectal SIV acquisition independent of epithelial inflammation. Although we were unable to define a clear role for uracil in delineating intra-rectal lentiviral susceptibility, these studies underscore the utility and feasibility of modeling metabolic contributions to lentiviral acquisition *in vivo*. Future studies may consider alternate delivery methods or vehicles to better understand the interplay between uracil, intestinal inflammation, and lentiviral susceptibility across the intra-rectal epithelium.

## Materials and methods

### Ethics statement

The NIAID Division of Intramural Research Animal Care and Use Program, as part of the NIH Intramural Research Program, approved all the experimental procedures (protocol LVD26E). The Program complies with all applicable provisions of the Animal Welfare Act and other federal statutes and regulations relating to animals. Animals were housed and cared for at the NIH Animal Center, under the supervision of the Association for the Assessment and Accreditation of Laboratory Animal Care (AAALAC)-accredited Division of Veterinary Resources and as recommended by the Office of Animal Care and Use Nonhuman Primate Management Plan. Husbandry and care met the standards set forth by the Animal Welfare Act, Animal Welfare Regulations, as well as The Guide for the Care and Use of Laboratory Animals (8^th^ Edition). The physical conditions of the animals were monitored daily. No animals met endpoint criteria as defined by: (a) loss of 25% body weight from weight when assigned to the protocol, (b) major organ failure or medical conditions unresponsive to treatment, (c) complete anorexia for 4 days or an inability to feed or drink sufficient nutrients to maintain body weight without assistance for 7 days, (d) distress vocalization unresponsive to treatment or intervention for 7 days, or (e) tumors arising from other than experimental means that grew in excess of 10% of body weight, impaired movement, or ulcerated. Following the cessation of this study, animals were enrolled in secondary protocols.

Animals in this study were exempt from contact social housing due to scientific justification, per the Institutional Animal Care and Use Committee protocol, and were housed in non-contact, social housing where primary enclosures consisted of stainless-steel primate caging. Animals were provided continuous access to water and offered commercial monkey biscuits twice daily as well as fresh produce, eggs, and bread products twice weekly and a foraging mix consisting of raisins, nuts, and rice thrice weekly. Enrichment to stimulate foraging and play activity was provided in the form of food puzzles, toys, cage furniture, and mirrors or television.

### Caco-2 cell culture

Caco-2 cells (ATCC HTB-37) were maintained in complete Dulbecco’s modified eagle medium (DMEM) - high glucose, high pyruvate DMEM supplemented with 10% fetal calf serum, 1% Penicillin-Streptomycin, and 1% nonessential amino acids - on standard, polystyrene tissue cultured T-75 flasks (Corning) at 37°C and 5% CO_2_. Cells were passaged at a 1:10 dilution when 60–80% confluence was reached with 0.05% Trypsin-EDTA. Media was changed three times a week. Cells were used at passage numbers 8 and 10.

Cells were either seeded at 8.4x10^4^ cells/well on standard Costar tissue cultured 6-well plates (Corning) in complete DMEM or seeded apically at 8x10^4^ cells/well onto 6.5 mm Transwell with a 0.4 um pore polycarbonate membrane insert (Corning) in complete DMEM and incubated at 37°C and 5% CO_2_. Plate-seeded Caco-2 cells were followed until 80% confluence was reached followed by 24 hours of vehicle-only (DMSO), TBHP, or uracil treatment. Transwell-seeded Caco-2 cells were treated 3 days post-seeding with either complete DMEM, or with DMSO, TBHP, uracil, or uridine treatment through day 17 post-seeding – treatment conditions were maintained for the study duration. Media was changed three times a week, in the morning. TEER (EVOM World Precision Instruments) readings were collected in Transwell-seeded Caco-2 cells at least five hours after media changes.

### *In vitro* ROS detection

Cells were stained with 10 uM 2’,7’- Dichlorodihydrofluorescein diacetate (DCFH-DA) (MilliporeSigma) in DMEM + 1% Penicillin-Streptomycin for 30 min at 37°C. Following stain, cells were washed once with 1 ml/well PBS and stimulated with either 0.1% DMSO, 100–400 uM TBHP (Thermo Fisher Scientific), 294 uM Uracil (Sigma), or 204 uM Uridine (Sigma) for 24 hours at 37°C and 5% CO_2_. Supernatants were collected for fluorescence assay, and cells were harvested for flow cytometry. Supernatants were collected, in duplicate, into 96-well black flat bottom polystyrene nonbinding surface microplates (Corning) at pre-determined timepoints following DCFH-DA staining. Fluorescence intensity of the ROS-reactive DCFH-DA derivative DCF was determined at excitation 488 nm and emission 528 nm on a Biotek Synergy H1 Microplate Reader (Gen5 v3.14.30). Caco-2 cells were stained for flow cytometry as described for *in vivo* cell staining, below.

### Animals and uracil treatment

Twelve healthy, adult, rhesus macaques (*Macaca mulatta)* were treated with oral uracil (n = 6) or uracil suppositories (n = 6), with sample size based on previous studies of experimental manipulations of disease progression in the rhesus macaque model [55]. Groups were stratified by weight, sex, and genotype (Mamu-A*001, -A*002, -B*008, and -B*017) and animals treated and sampled as mixed populations [56]. Animals treated with oral uracil (MP Biomedicals) received 125 mg/m^2^ body-surface area, twice-daily sprinkled into meals, until confirmed infection. Following anesthesia with either 3mg/kg of Telazol or 10–15mg/kg Ketamine, animals receiving colonic uracil suppositories received 1500mg of uracil thrice-weekly by video-guided endoscopy. For colonic suppository, uracil was compounded at 10% (w/w) in cocoa butter (MP Biomedicals) at 37°C in individual aliquots. Aliquots were frozen at -20°C immediately after preparation and thawed back to 37°C by water bath immediately prior to administration. Under anesthesia with either 3mg/kg of Telazol or 10–15mg/kg Ketamine, all animals were challenged with 4 TCID_50_ SIV_mac239X_ i.r. every 14 days as outlined in Figs 1 and 5. Contemporaneous challenge controls for the oral uracil treated animals included all controls from Ortiz et al. [18], and for the uracil suppository treated animals from Ortiz et al. [57].

### Sample collection

Blood and intestinal biopsies were collected longitudinally. Sampling occurred in random order. If biopsies or challenge timepoints fell on a suppository day, suppositories were skipped for that specific day, to avoid technical interference with sample collection or challenge. Neither the investigators nor the animal handlers were blinded to group allocation to ensure multi-lateral supervision of design and palliative treatment. Animals were sedated with Telazol at 3–4 mg/kg i.m. and were further anesthetized with isoflurane gas by intubation, to effect. Successful anesthetization was monitored by response to stimuli.

Whole blood was collected into EDTA-containing tubes. For rectal biopsies, fecal material was removed from the rectum and biopsies obtained with biopsy forceps. Jejunal biopsies were acquired by video-guided endoscopy, with samples collected by biopsy forceps. 10 intestinal, pinch biopsies were obtained per animal at each timepoint. All biopsies were transported in RPMI and washed twice in PBS prior to processing.

Three of 10 intestinal pinch biopsies were transferred to PowerBead tubes (Qiagen, Hilden, Germany) and homogenized in 1 mL TRIzol (Thermo Fisher Scientific, Waltham, MA) at room temperature on a Precellys 24 homogenizer (Bertin Technologies, Rockville, MD) at 5000 revolutions per minute in 4 successive 20 sec intervals. TRIzol-preserved homogenates were stored at -80°C until accession. Plasma was isolated from whole blood by centrifugation. Mononuclear cells were isolated from blood by Ficoll gradient centrifugation and from whole rectal and jejunal pinch biopsies by mechanical disruption through a 70-µm cell strainer; IEL and LPL fractions were not separately isolated. Approximately 1mL of RM stool was collected fresh from each animal by inserting a sterile swab 2 cm into the rectum and spinning to collect available sample. Collected feces were snap-frozen and stored at -80°C until accession.

### Immune phenotyping and functional assessment

Polychromatic flow cytometry was performed on stained mononuclear cells, using a BD LSRII (FACSDiva v8.0.1) or Cytek Aurora 5L (Cytek Biosciences, SpectroFlow v3.0.3). Antibodies against the following antigens were used for staining at predetermined concentrations: CD3 (SP34–2) AlexaFluor (AL)-700, CD4 (OKT4) Brilliant Ultra Violet (BUV)-496, CD20 (2H7) APC-H7, CD20 (L27) BUV615, CD28 (CD28.2) PE-CF594, CD45 (D058-1283) BV786, and Ki-67 (B56) BUV395 or FITC from BD (Franklin Lakes, NJ); CD4 (OKT4) eFluor-450, CD8 (SK1) PerCP-eFluor-710, IFNγ (4S.B3) eFluor-450, IL-17 (eBio64DEC17) PE-Cy7, and IL-22 (IL22JOP) APC from Thermo Fisher Scientific; CD31 (WM59) APC-Cy or BV711, CD95 (DX2) PE-Cy5, EPCAM (9C4) PE-Dazzle, and TNFα (Mab11) BV605 from Biolegend (San Diego, CA). ROS production was assessed by staining with CellROX Green (Thermo Fisher Scientific). To assess cytokine potential, cells were stimulated for 16 hours with 2.5ng/mL PMA and 1 ug/mL ionomycin in 1mL of R10 (RPMI supplemented to 10% fetal calf serum,100 U/ml penicillin, 100 μg/ml streptomycin sulfate, 1.7 mM sodium glutamate) with 10ug/mL Brefeldin A. Cell viability was assessed using the Live/Dead Aqua Fixable Dead Cell Stain (Thermo Fisher Scientific). Cells were permeabilized with the eBioscience Intracellular Fixation & Permeabilization Buffer Set (Thermo Fisher Scientific) prior to intracellular staining. Cell populations were defined as in S2 Fig, with positive/negative gating based on clearly grouped populations, historical-determined expression, and the use of internal controls. A threshold of at least 100 collected events in the parent population was used for all subset expression analyses [FlowJo (BD, v10.10.0) or OMIQ (Dotmatics, Boston, MA)]. Radar plots were generated from fold-change expression data using ggradar (v0.2) in RStudio (v2023.12.1 + 402) using R (v4.3.3).

### ELISAs

Concentrations of Calprotectin, iFABP2, sCD14, and zonulin were quantified using commercially available ELISA kits [Eagle Bioscience (Amherst, MA) #CAL35-K01, My Biosource (San Diego, CA) #MBS740424, R&D (Minneapolis, MN) #DC140, and Alpco (Salem, NH) # 30-ZONSHU-E01, respectively]. iFABP2, sCD14, and zonulin were quantified from plasma according to the manufacturers’ protocols. Calprotectin was quantified from fecal extracts with modifications from the manufacturer’s fecal extraction protocol. Fecal aliquots were thawed to 4°C, weighed (range 35–208mg), and homogenized with 49x weight-volume extraction buffer. Samples were homogenized at room temperature on a microplate shaker set to maximum rotation for 50 minutes. Samples were filtered at 70 um and the filtrate centrifuged at 3000 x*g* for 5 minutes. The resultant supernatant was assayed per the manufacturer’s protocol.

### RNA extraction, quantification, and assessment

For intestinal transcript quantification TRIzol-preserved samples were thawed and treated with 200μL chloroform to separate nucleic acid into an aqueous phase. Following separation, total RNA was isolated from the aqueous phase using the MagMAX-96 total RNA isolation kit (Thermo Fisher Scientific) per the manufacturer’s protocol. RNA concentration and purity (A260/280 ≥ 1.8) were assessed by spectrophotometer and normalized to 50–100 ng/μL in PCR-grade H_2_O. For transcript quantification by NanoString, preparation, hybridization, and detection of RNA samples were carried out by following the NanoString manufacturer’s instructions (NanoString Technologies, Seattle, WA) using the nCounter NHP Immunology Panel. Read pre-processing was performed using the nSolver (v4.0) analysis system (NanoString Technologies). Reads from NanoString were normalized to internal positive and negative controls and housekeeping genes. Transcriptome variability was normalized and calculated using the prcomp function and visualized by PCA using the ggbiplot package (v0.6.2), in RStudio using R. The identification and normalization of differentially abundant transcripts was determined by DESeq2 [58]. DESeq2 (v1.42.1) was run utilizing the Wald test with Benjamini-Hochberg for false-discovery. Venn Diagrams were created with ggVennDiagram (v1.5.2) in RStudio. Pathway enrichment was determined by Gene Set Enrichment Analysis (GSEA; Broad Institute, Inc., Massachusetts Institute of Technology, and Regents of the University of California; Desktop V3.0) against the ‘h.all.v2025.1.Hs.symbols.gmt’ dataset.

For individual transcript quantification, cDNA was generated using the SuperScript III or IV reverse transcriptase kits (Thermo Fisher Scientific). qRT-PCR for *DUOX2* was performed using transcript-specific Gene Expression Assays (Thermo Fisher Scientific) with TaqMan Fast Advanced Master Mix and were normalized to housekeeping gene (monkey) beta-glucuronidase (mGusB) utilizing a custom-designed mGusB TaqMan Gene Expression Assay (Thermo Fisher Scientific). Custom mGusB primers and probe as follows: forward primer 5’-CTCATTTGGAATTTTGCCGATT-3’, reverse primer 5’-CCGAGTGAAGATCCCCTTTTTA-3’, and probe 5’-TGAACAGTCACCGACGAGAGTGCTGG-3’. Relative transcript measurement was assessed in triplicate on the Applied Biosystems StepOne Plus Real Time PCR System with StepOne Software (Thermo Fisher Scientific, v2.0) or the QuantStudio 3 System with the QuantStudio Design and Analysis Software (Thermo Fisher Scientific, v1.5.2), using the recommended TaqMan Fast Advanced cycling parameters.

### Mass spectrometry

Metabolites were quantified from stool by untargeted metabolomics by Gigantest, Inc. Stool samples were homogenized in a solvent mixture of 99% LC-MS-grade acetonitrile and 1% formic acid using a multi-probe sonicator. Homogenized samples were transferred to an extraction plate containing a sorbent filter designed for protein precipitation and phospholipid removal. The metabolite solution was passed through the sorbent filter using a Positive Pressure-96 processor, collecting the purified metabolite solution in an MS plate while retaining proteins and phospholipids in the sorbent filter. Extracted solutions were dried down to 150 µL for acquisition. Data was acquired using a Thermo Fisher Scientific IQ-X Mass Spectrometer with a Vanquish UPHLC system. A reversed-phase chromatography method, employing a Discovery HSF5 column (Sigma), was used with a 9-minute run time; 7 minutes for data acquisition and 2 minutes for column equilibration. Samples were maintained at 4°C throughout the chromatography system and injected with a volume of 2μL - the column was kept at 35°C. The mobile phases consisted of 0.1% formic acid in MS-water (aqueous) and 0.1% formic acid in acetonitrile (organic). Hydrophilic Interaction Liquid Chromatography (HILIC) was also used with the ACQUITY PRM BEHAmide column and VanGuard FIT Cartridge (Waters) with a 15-minute run time; 13.5 minutes for data acquisition and 1.5 minutes for column equilibration. Similar to the reverse-phase chromatography, samples were kept at 4°C during acquisition and injected with a volume of 2μL - the column was kept at 35°C. Mobile phases consisted of 95:5 20mM ammonium acetate + 20mM ammonium hydroxide pH 9 in water and 100% acetonitrile. Using Gigantest’s Laboratory Information Management System (LIMS) in combination with Python and R, metabolites were identified based on retention times previously determined from pure standards, as well as using mass accuracy. The final metabolite intensity data was obtained by integrating the area under each chromatographic peak and then normalizing the values to the sample weight. Each chromatographic peak was manually reviewed after applying machine learning algorithms to ensure accurate peak integration. Two samples were removed from analysis – one that was identified as an outlier by the 1.5xIQR rule and its matched pair.

### 16S isolation and analysis

16S rDNA was isolated and sequenced as previously described [59]. Raw Illumina FASTQ files were first demultiplexed using a custom Python script. Returned paired-end FASTQ reads were filtered and processed using the DADA2 package (v1.30.0) in RStudio to infer ASVs at a 99% identity threshold using the Silva database (v138.1). Before quality trimming 5,812,993 reads were included in 36 samples with an average of 161,472 reads per sample. Reads were trimmed to 225 bp (forward) or 200 bp (reverse) and filtered to exclude sequences with degenerate bases (N), more than 2 expected errors (maxEE), or chimerism. DADA2 quality trimming resulted in 3,683,389 reads for all the samples with an average of 102,316 reads per sample. 1 sample with less than 1000 reads was omitted from further analysis. ASVs that could not be classified or that were identified as non-Bacteria, Cyanobacteria, or Chlorobiota, were removed from further consideration as were resultant genera at less than 1% prevalence or phyla with no genera diversity. Differentially abundant taxa were identified by ANCOM-BC or ANCOM-BC2 (v2.4.0) in RStudio [60]. Fully analyzed 16S miSeq data (n = 35) are deposited in the NCBI Sequence Read Archive (SRA) under project number PRJNA1415728.

### VL and T/F assessment

Plasma viral RNA levels and assessment of T/F lineages were determined as previously described [18,27,61].

### Statistical analyses

Caco-2 monolayer data was assessed by one-way ANOVA. As appropriate, unpaired or paired, one- or two-way t-tests and mixed effects model (REML) were used in statistical analyses of ELISA-derived concentrations, lymphocyte phenotype and function frequencies, bacterial α-diversity, mass spectrometry, acquired T/F variants, and viral loads (Prism v9.0, GraphPad Software Inc.). Normalization and statistical evaluation of the NanoString-assessed transcriptome was determined by DESeq2 [58], utilizing the Wald test with Benjamini-Hochberg for false-discovery. Pathway enrichment was determined by GSEA which includes the Kolmogorov-Smirnov test for p-value determination. NanoString-derived transcript clustering and bacterial β-diversity were evaluated by adonis (PERMANOVA) in RStudio. Differentially abundant bacterial ASVs were identified by ANCOMB-BC in RStudio. Potential significance for the number of challenges required for infection were assessed by Mantel-Cox. T/F correlations were assessed by Spearman correlation (Prism). No data that met minimum threshold requirements as outlined in the Methods were excluded.

## Supporting information

S1 Fig Uracil treatment does not induce epithelial cell ROS *in vitro.* (A) Fluorescence intensity of ROS probe DCF in treated, plated Caco-2 cell supernatants. (B-C) Frequency of Ki67-expressing (B) and viable (C) treated, plated Caco-2 cells. (D) Fluorescence intensity of ROS probe DCF in treated, transwell-grown Caco-2 cell supernatants. (E-F) Frequency of Ki-67-expressing (E) and viable (F), treated, transwell-grown Caco-2 cells. (G) Longitudinal TEER readings in transwell-grown Caco-2 cells. Bar heights represent mean + /- SEM. Each data point in A-F represents one technical replicate; datapoint and line in G represent mean of technical replicates. All significance assessed by one-way ANOVA.
(TIF)

S2 Fig Longitudinal flow cytometry gating strategy. Flow cytometry gating defining memory CD4+ and CD8 + T-cells (A), cytokine and activation markers on CD4+ and CD8 + TM (B), and epithelial cell ROS staining (C).
(TIF)

S3 Fig Oral uracil treatment perturbs aspects of systemic immunity. (A) Radar plot depicting fold-change from Pre-treatment expression of markers/cytokines in CD8 + TM at indicated sites in oral uracil-treated macaques. Cytokine expression measured in response to *ex vivo* PMA/ionomycin stimulation. p-values are color-coded by tissue site and derived from non-normalized expression values. (B) PCA considering NanoString-enumerated Jej transcripts from oral uracil-treated macaques Pre- and Post-treatment. (C) Volcano plot depicting the fold-change expression of NanoString-enumerated Jej transcripts at the Post-treatment timepoint. Transcripts are color-coded by significance. (D) ROS MFI in Jej epithelial cells. (E) Normalized counts of Jej gene transcripts identified as differentially expressed within both RB and Jej homogenates. Significance in A assessed by paired two-way t-test on frequency-of-CD8 + TM expression values, in B by adonis (PERMANOVA), in C and E by DESeq2, and in D by paired, one-way t-test.
(TIF)

S4 Fig Suppository uracil treatment perturbs aspects of systemic immunity. (A) ROS MFI in Jej epithelial cells. (B) Radar plot depicting fold-change from Pre-treatment expression of markers/cytokines in CD8 + TM at indicated sites in uracil suppository-treated macaques. Cytokine expression measured in response to *ex vivo* PMA/ionomycin stimulation. p-values evaluate the difference of expression values at the Pre versus Post timepoints within each tissue and are color-coded by tissue site. (C) PCA analysis considering NanoString-enumerated RB (left) and Jej (right) transcripts from uracil suppository-treated macaques Pre- and Post-treatment. (D) Volcano plot depicting the fold-change expression of NanoString-enumerated Jej transcripts at the Post-treatment timepoint. Transcripts are color-coded by significance. Significance in A assessed by paired, one-way t-test, in B by paired two-way t-test on frequency-of-CD8 + TM expression values, in C by adonis (PERMANOVA), and in D by DESeq2.
(TIF)

S5 Fig Uracil suppositories modestly perturb the fecal microbiome. (A) Relative fecal bacterial abundance of uracil suppository-treated animals at Pre-treatment, Post-treatment, and on the day of the first SIV challenge (Chall). Taxa are color-grouped by phylum and individually shaded by family. (B) Fold-change frequencies of fecal ASVs identified by ANCOM-BC as longitudinally, differentially abundant at the time of challenge relative to Pre-treatment. Data show the natural log fold-change, color-grouped by phylum and individually shaded by family. (C) Richness (Observed OTUs) and Shannon indices of fecal bacterial a-diversity across the pre-infection period. (D) PCoA of unweighted and weighted UniFrac measures of fecal bacterial b-diversity. Bar segments in A denote mean relative abundance. Lines in C denote mean a-diversity. Significance in A-B calculated by ANCOM-BC, in C by paired two-way t-tests, and in D by adonis (PERMANOVA).
(TIF)

S1 Dataset Caco-2 *in vitro* data. (XLSX)

S2 Dataset Targeted assay data. (XLSX)

S3 Dataset NanoString data. (XLSX)

S4 Dataset Microbiome data. (XLSX)
